# Supplementary figures and images for: A retrospective study of vector borne disease prevalence among anemic dogs in North Carolina
Source: PLoS One. 2023 Nov 8;18(11):e0293901. doi: 10.1371/journal.pone.0293901 (PMC10631695; doi:10.1371/journal.pone.0293901)

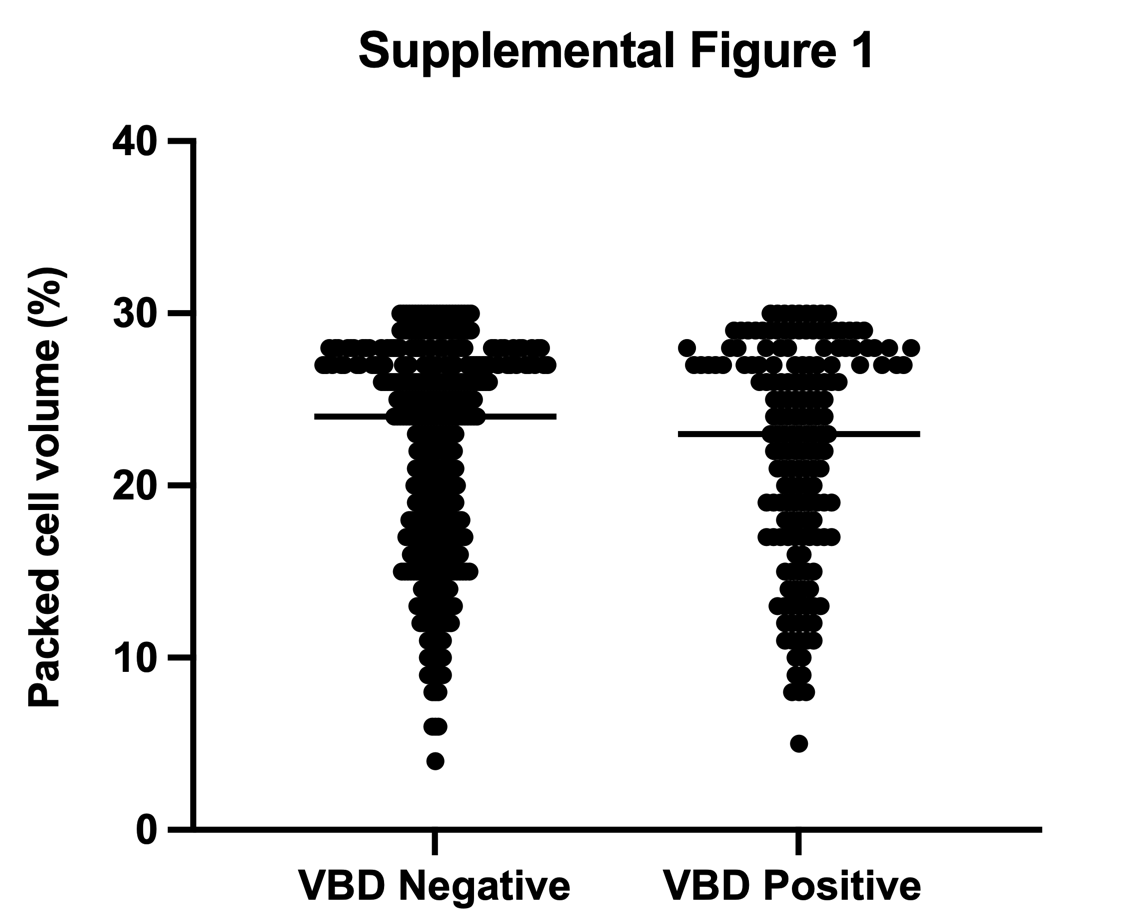

Supplement: S1 Fig — The packed cell volume (y axis) of dogs testing positive or negative for VBD (x axis) is shown. Each dot represents an individual dog. The bar represents the median packed cell volume for each group (test positive median PCV 23% (range 5–30%); test negative median PCV 24% (range 4–30)). (TIFF) [file pone.0293901.s001.tiff]

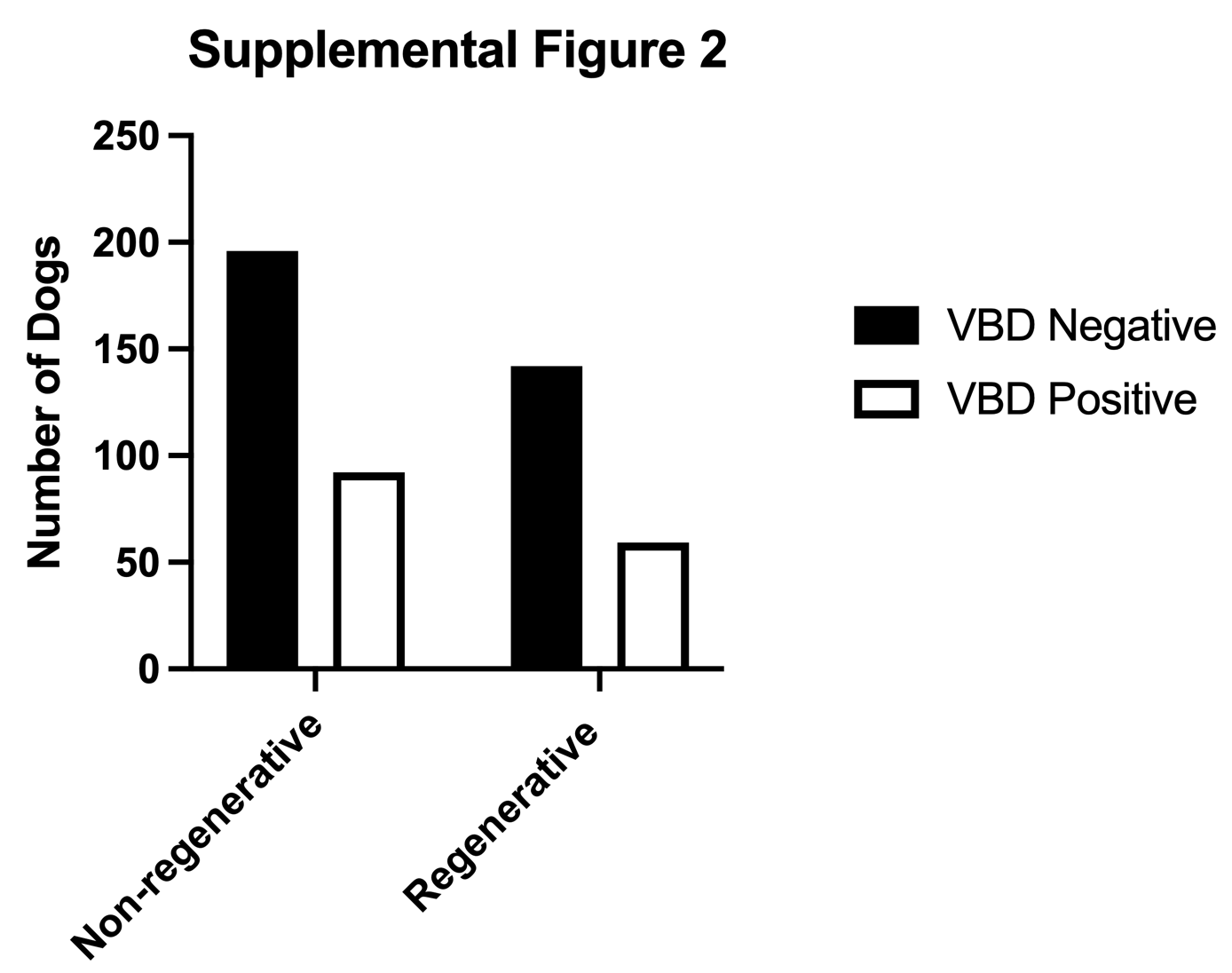

Supplement: S2 Fig — Regeneration was defined as a reticulocyte count greater than or equal to 60,000 cells/uL (x-axis). The number of dogs in each group is shown on the y-axis. The black bars represent dogs testing negative for all VBD while the white bars represent dogs testing positive for one or more VBD. (TIFF) [file pone.0293901.s002.tiff]
