# Supplementary material for: A retrospective study of vector borne disease prevalence among anemic dogs in North Carolina
Source: PLoS One. 2023 Nov 8;18(11):e0293901. doi: 10.1371/journal.pone.0293901 (PMC10631695; doi:10.1371/journal.pone.0293901)
Supplement: S3 Table — (DOCX) [file pone.0293901.s005.docx]

**Supplemental Table 1: Number of dogs testing positive for exposure to or infection with multiple vector borne diseases**

| Co-infections/co-exposures | Number of dogs testing positive for exposure/infection |
| --- | --- |
| *Ehrlichia* spp*., Rickettsia* spp*.* | 14 |
| *Borrelia burgdorferi, Rickettsia* spp*., Bartonella* spp*.* | 4 |
| *Borrelia burgdorferi* spp*., Rickettsia* spp*.* | 4 |
| *Ehrlichia* spp*., Rickettsia* spp*., Bartonella* spp*.* | 3 |
| *Babesia* spp*., Rickettsia* spp*.* | 3 |
| *Borrelia burgdorferi, Babesia* spp*., Rickettsia* spp*.* | 2 |
| *Mycoplasma* spp*., Borrelia burgdorferi, Ehrlichia* spp*.* | 2 |
| *Ehrlichia* spp*., Bartonella* spp*.* | 2 |
| *Rickettsia* spp*., Bartonella* spp*.* | 2 |
| *Borrelia burgdorferi, Dirofilaria immitis, Ehrlichia* spp*., Rickettsia* spp*., Bartonella* spp*.* | 1 |
| *Borrelia burgdorferi, Ehrlichia* spp*., Anaplasma* spp*., Dirofilaria immitis* | 1 |
| *Borrelia burgdorferi, Anaplasma* spp*., Rickettsia* spp*.* | 1 |
| *Mycoplasma* spp*., Ehrlichia* spp*., Rickettsia* spp*.* | 1 |
| *Ehrlichia* spp*., Borrelia burgdorferi, Rickettsia* spp*.* | 1 |
| *Mycoplasma* spp*., Rickettsia* spp*., Bartonella* spp*.* | 1 |
| *Anaplasma* spp*., Rickettsia* spp*., Bartonella* spp*.* | 1 |
| *Borrelia burgdorferi, Bartonella* spp*.* | 1 |
| *Mycoplasma* spp*., Ehrlichia* spp*.* | 1 |
| *Mycoplasma* spp*., Dirofilaria immitis* | 1 |
| *Babesia* spp*., Bartonella* spp*.* | 1 |
| *Ehrlichia* spp*., Bartonella* spp*.* | 1 |
| *Babesia* spp*., Ehrlichia* spp*.* | 1 |
