# Supplementary material for: A retrospective study of vector borne disease prevalence among anemic dogs in North Carolina
Source: PLoS One. 2023 Nov 8;18(11):e0293901. doi: 10.1371/journal.pone.0293901 (PMC10631695; doi:10.1371/journal.pone.0293901)
Supplement: S4 Table — (DOCX) [file pone.0293901.s006.docx]

|  | Number of positive tests with spherocytes present /total number of positive tests examined | Number of tests with agglutination present / total number of positive tests examined | Number of positive Coombs tests/number of Coombs tests performed |
| --- | --- | --- | --- |
| VBD positive | 21/180 (11.7%) | 26/180 (14.4%) | 9/32 (28.1%) |
| VBD negative | 100/412 (24.3%) | 79/412 (19.2%) | 16/69 (23.2%) |
