# Supplementary material for: A retrospective study of vector borne disease prevalence among anemic dogs in North Carolina
Source: PLoS One. 2023 Nov 8;18(11):e0293901. doi: 10.1371/journal.pone.0293901 (PMC10631695; doi:10.1371/journal.pone.0293901)
Supplement: Attachment — Submitted filename: Response to reviewers PLOS ONE.docx [file pone.0293901.s007.docx]

September 23rd, 2023

Re: Manuscript ID PONE-D-23-1484, entitled “A retrospective study of vector borne disease prevalence among anemic dogs in North Carolina.”

Dear Dr. Kamani and reviewers,

We were pleased to hear that our manuscript, entitled “A retrospective study of vector borne disease prevalence among anemic dogs in North Carolina," may be acceptable for publication in *PLOS ONE* pending revision, and we would like to thank you for your efforts on our behalf. We appreciate the reviewer’s detailed and constructive comments regarding our article and have addressed these comments in the response below and in the revised manuscript, as indicated. We believe that our manuscript is substantially improved after making the suggested edits.

Please do not hesitate to contact us with any questions you may have regarding our manuscript.

Thank you for your time and consideration,

Katie Anderson, Allison Kendall, and Adam Birkenheuer

***Journal Requirements:***

*When submitting your revision, we need you to address these additional requirements.*

1) *Please ensure that your manuscript meets PLOS ONE's style requirements, including those for file naming. The PLOS ONE style templates can be found at*
   [*https://journals.plos.org/plosone/s/file?id=wjVg/PLOSOne_formatting_sample_main_body.pdf*](https://journals.plos.org/plosone/s/file?id=wjVg/PLOSOne_formatting_sample_main_body.pdf)  *and* 
   [*https://journals.plos.org/plosone/s/file?id=ba62/PLOSOne_formatting_sample_title_authors_affiliations.pdf*](https://journals.plos.org/plosone/s/file?id=ba62/PLOSOne_formatting_sample_title_authors_affiliations.pdf)

We have edited our manuscript to comply with style requirements. Specifically, we have changed our references to comply with the requirements by using brackets instead of parentheses. We have also submitted our files with the correct labeling. We apologize for overlooking these requirements in our previous submission.

2) *Thank you for including your ethics statement:  "Due to the retrospective nature of this manuscript, no IACUC approval was needed.".*

*To comply with PLOS ONE submissions requirements, please provide the following information in the Methods section of the manuscript and in the “Ethics Statement” field of the submission form (via “Edit Submission”):  
*  Please indicate whether an animal research ethics committee prospectively approved this research or granted a formal waiver of ethics approval.*  Please enter the name of your Institutional Animal Care and Use Committee (IACUC) or other relevant ethics board. Also include an approval number if one was obtained.
*   If anesthesia, euthanasia, or any kind of animal sacrifice is part of the study, please include briefly in your statement which substances and/or methods were applied.*

*For additional information about PLOS ONE submissions requirements for ethics oversight of animal work, please refer to* [*http://journals.plos.org/plosone/s/submission-guidelines#loc-animal-research*](http://journals.plos.org/plosone/s/submission-guidelines#loc-animal-research)

*Once you have amended this/these statement(s) in the Methods section of the manuscript, please add the same text to the “Ethics Statement” field of the submission form (via “Edit Submission”).*

The data for this study were acquired from historic, clinical patients at NC State University and thus meet the following conditions: “activities in which a veterinarian is providing routine clinical services to a privately owned animal.” These conditions do not require IACUC approval at NC State University. All patients admitted to NC State University are required to sign a consent form that allows the use of anonymized patient data for research purposes. Please see the following website for additional details: <https://research.ncsu.edu/administration/compliance/research-compliance/iacuc/iacuc-procedures-and-guidance/guidance-on-activities-requiring-iacuc-approval/>.

The following statement has been added to lines 149-153 of the methods section of the manuscript:

“**Ethics statement**

This manuscript was retrospective in nature and utilized data from clinical patients. All data was anonymized prior to use. Given the retrospective nature of this manuscript, prospective approval from the NC State Institution Animal Care and Use Committee (IACUC) was not necessary and a formal waiver of ethics approval was not obtained.”

*3. Please include captions for your Supporting Information files at the end of your manuscript, and update any in-text citations to match accordingly. Please see our Supporting Information guidelines for more information:* [*http://journals.plos.org/plosone/s/supporting-information*](http://journals.plos.org/plosone/s/supporting-information)*.*

The captions for the Supporting Information have been added to the end of the manuscript according to the guidelines (see lines 413-429).

***Reviewers' comments:

Reviewer's Responses to Questions***

***Comments to the Author*** *1. Is the manuscript technically sound, and do the data support the conclusions?

The manuscript must describe a technically sound piece of scientific research with data that supports the conclusions. Experiments must have been conducted rigorously, with appropriate controls, replication, and sample sizes. The conclusions must be drawn appropriately based on the data presented.*

*Reviewer #1: Yes*

*2. Has the statistical analysis been performed appropriately and rigorously?*

*Reviewer #1: N/A*

*3. Have the authors made all data underlying the findings in their manuscript fully available?

The* [*PLOS Data policy*](http://www.plosone.org/static/policies.action#sharing) *requires authors to make all data underlying the findings described in their manuscript fully available without restriction, with rare exception (please refer to the Data Availability Statement in the manuscript PDF file). The data should be provided as part of the manuscript or its supporting information, or deposited to a public repository. For example, in addition to summary statistics, the data points behind means, medians and variance measures should be available. If there are restrictions on publicly sharing data—e.g. participant privacy or use of data from a third party—those must be specified.*

*Reviewer #1: Yes*

*4. Is the manuscript presented in an intelligible fashion and written in standard English?

PLOS ONE does not copyedit accepted manuscripts, so the language in submitted articles must be clear, correct, and unambiguous. Any typographical or grammatical errors should be corrected at revision, so please note any specific errors here.*

*Reviewer #1: Yes*

*5. Review Comments to the Author

Please use the space provided to explain your answers to the questions above. You may also include additional comments for the author, including concerns about dual publication, research ethics, or publication ethics. (Please upload your review as an attachment if it exceeds 20,000 characters)*

*Reviewer #1: This is an interesting and well written paper. It includes important information relating to vector-borne diseases of dogs in North Carolina. I suggest accepting for publication following correcting the below comments.*

*Major comments
Can the authors explain why cases from 2019-2022/2023 were excluded from the study? I am asking so as 4-5 years, under current global changes, can affect the epidemiologic picture?*

We began preparing this retrospective manuscript in 2019, and therefore chose to include cases prior to this date. However, we agree with the reviewer that the epidemiologic picture may change over time, and we intend to re-evaluate the data every 5-10 years to monitor for significant changes.

*L 14-141 – " Primers, PCR conditions, and negative and positive controls were used as previously described (11–14)." Authors should include the list of primers, PCR conditions, and negative and positive controls used at least as a supplemental material. Readers, researchers and reviewers should be able to evaluate the tests' protocols and do not have to look for these important protocols in other articles.*

We have included all PCR primers, reaction conditions, and positive/negative controls in a supplemental file (S1 Table). While creating this file, we decided to include several additional references to more comprehensively cite our protocols (see references 11-21). In addition, the following section has been added to lines 142-152 of the materials and methods section:

“Primers, PCR conditions, and negative and positive controls were used as previously described [11–22]. Briefly, DNA was extracted from stored canine ethylenediaminetetraacetic acid (EDTA) whole blood samples using a commercially available MagAttract DNA blood kit (Qiagen Inc, Chatsworth, CA) according to the manufacturer’s instructions. For all samples, the DNA concentration was quantified by spectrophotometry, and the absence of PCR inhibitors was demonstrated by amplification of a fragment of the glyceraldehyde-3-phosphatase dehydrogenase gene. PCR screening was performed as previously described [11-22]; a comprehensive list of the PCR primers, reaction volumes, and reaction conditions used is available in supplemental table 1. Products were analyzed by 2% agarose gel electrophoresis containing 0.2ug ethidium bromide/mL under ultraviolet light. Canine DNA from a healthy subject was used as a PCR negative control. Positive controls are listed in supplemental table 1.”

*Minor comments
L 109 – should be cells/ul
L 139 – should be Anaplasma spp.
L 170 and legends of Figures 2 & 3 – should be disease (and not diseases)
L 289, 291 and all over the text – spp. should not appear in italics.
Table 4 – lines representing Bartonella spp. PCR and Rickettsia spp. PCR should be removed from the table or the legend should be corrected (as currently includes:"…. dogs testing positive for individual vector borne diseases".
L 414 – Ref 17 – pages should be added.
Supplemental Table 1 – all spp. should NOT appear in italics.*

We would like to thank the reviewer for catching these errors. All minor comments have been corrected within the revised manuscript.

*6. PLOS authors have the option to publish the peer review history of their article (*[*what does this mean?*](https://journals.plos.org/plosone/s/editorial-and-peer-review-process#loc-peer-review-history)*). If published, this will include your full peer review and any attached files.

If you choose “no”, your identity will remain anonymous but your review may still be made public.*

***Do you want your identity to be public for this peer review?*** *For information about this choice, including consent withdrawal, please see our* [*Privacy Policy*](https://www.plos.org/privacy-policy)*.*

*Reviewer #1: No*
