# Supplementary material for: A retrospective study of vector borne disease prevalence among anemic dogs in North Carolina
Source: PLoS One. 2023 Nov 8;18(11):e0293901. doi: 10.1371/journal.pone.0293901 (PMC10631695; doi:10.1371/journal.pone.0293901)
Supplement: Attachment — Submitted filename: Response to reviewers PLOS ONE_Oct.docx [file pone.0293901.s008.docx]

October 19, 2023

Re: Manuscript ID PONE-D-23-1484, entitled “A retrospective study of vector borne disease prevalence among anemic dogs in North Carolina.”

Dear Dr. Kamani and reviewers,

We were pleased to hear that our manuscript, entitled “A retrospective study of vector borne disease prevalence among anemic dogs in North Carolina," may be acceptable for publication in *PLOS ONE* pending revision, and we would like to thank you for your efforts on our behalf. We appreciate the reviewer’s detailed and constructive comments regarding our article and have addressed these comments in the response below and in the revised manuscript, as indicated. We believe that our manuscript is substantially improved after making the suggested edits.

Please do not hesitate to contact us with any questions you may have regarding our manuscript.

Thank you for your time and consideration,

Katie Anderson, Allison Kendall, and Adam Birkenheuer

***Additional Editor Comments:

Reviewer's Responses to Questions***

*The authors are invited to kindly address the following comments:
Line 137 Ehrlichia spp add full stop after spp. ‘’.’’ Ehrlichia spp’’.’’ and several places in the manuscript after’’ spp.’’ especially Table 3,4* Thank you, this has been edited throughout the manuscript  *Line 137 what is Ehrlichia spp ‘’dot’’?* This has been changed to ‘positive spot’ to describe the positive result of Ehrlichia on a SNAP 4Dx  *Line 159: Anaplasma spp. not : Anaplasmosis spp. please correct it* This has been corrected.  *Line 183-184 should not be italic.* This has been corrected.  *Line 195. D. immitis instead of Dirofilaria immitis* Thank you, this has been edited  *Line 201. 288. B. canis not Babesia canis’’.’’ Remove ‘’.’’* This has been corrected.  *Line 202, 289. B. gibsoni not Babesia gibsoni* This has been corrected  *Line 211 correct------2 dogs ‘’with’’ infected with----* Thank you, this has been corrected  *Tables 2,3,4. italicize genus and species names appropriately* This has been edited in the tables  *Line 218 abbreviations should not be italicized.* This is corrected  *Line 248 remove one ‘’.’’---- anaemia.”.””* Thank you, this has been edited  *Line 303-304-Does this mean dogs examined at the hospital or are they dogs kept at the Institution? Rephrase* This has been rephrased  *Lines 332-334- Recast the sentence appropriately* This sentence has been rephrased
